# Developing and applying a 'living guidelines' approach to WHO recommendations on maternal and perinatal health

**DOI:** 10.1136/bmjgh-2019-001683

**Published:** 2019-08-19

**Authors:** Joshua P Vogel, Therese Dowswell, Simon Lewin, Mercedes Bonet, Lynn Hampson, Frances Kellie, Anayda Portela, Maurice Bucagu, Susan L Norris, James Neilson, Ahmet Metin Gülmezoglu, Olufemi T Oladapo

**Affiliations:** 1 UNDP/UNFPA/UNICEF/WHO/World Bank Special Programme of Research, Development and Research Training in Human Reproduction (HRP), Department of Reproductive Health and Research, World Health Organization, Geneva, Switzerland; 2 Maternal and Child Health Program, Burnet Institute, Melbourne, Victoria, Australia; 3 Cochrane Pregnancy and Childbirth, University of Liverpool, Liverpool, UK; 4 Division of Health Services and Centre for Informed Health Choices, Norwegian Institute of Public Health, Oslo, Norway; 5 Health Systems Research Unit, South African Medical Research Council, Cape Town, South Africa; 6 Cochrane Effective Practice and Organisation of Care, Norwegian Institute of Public Health, Oslo, Norway; 7 Department of Maternal, Newborn, Child and Adolescent Health, World Health Organization, Geneva, Switzerland; 8 Department of Information, Evidence and Research, World Health Organization, Geneva, Switzerland

**Keywords:** clinical guidelines, maternal and perinatal health, living guidelines, living recommendations, living systematic reviews, recommendations, world health organization

## Abstract

How should the WHO most efficiently keep its global recommendations up to date? In this article we describe how WHO developed and applied a ‘living guidelines’ approach to its maternal and perinatal health (MPH) recommendations, based on a systematic and continuous process of prioritisation and updating. Using this approach, 25 new or updated WHO MPH recommendations have been published in 2017–2018. The new approach helps WHO ensure its guidance is responsive to emerging evidence and remains up to date for end users.

**Summary box**

- Over the past decade, WHO has issued over 400 maternal and perinatal health (MPH) recommendations for global use, and the size of this portfolio presents a major challenge to ensuring that all recommendations are up to date.
- A dynamic ‘living guidelines’ approach has been developed and applied to respond more rapidly to new, important evidence that may affect specific WHO recommendations in MPH.
- The new approach uses an evidence-informed, consultative prioritisation process, rapid updating of prioritised systematic reviews and electronic consultations with ‘living guidelines’ panels.
- Using this approach enables WHO to efficiently use resources to execute its global mandate on normative guidance for MPH.
- Other guideline development organisations can also adapt this approach to facilitate more rapid and efficient updating of recommendations.

## Introduction

WHO is the normative entity of the United Nations responsible for developing and disseminating evidence-based guidance on health issues. Since the establishment of the WHO Guidelines Review Committee (GRC) in 2007 to oversee the quality of WHO’s guidelines, WHO has issued 13 maternal and perinatal health (MPH) guidelines containing 295 clinical and health systems recommendations,^1–13^ a health system guideline on task-shifting in reproductive, maternal and newborn health with 119 recommendations (OptimizeMNH guideline),^14^ and interim guidance on pregnancy management in the context of Zika virus infection.^15^ Like other organisations that develop guidelines, WHO faces a major challenge in keeping these recommendations up to date as the evidence base evolves and expands, the needs of end users change, and new public health priorities emerge.^16–19^

There are no rules on how frequently guidelines produced by WHO should be updated, though the *WHO Handbook for Guideline Development* indicates that guidelines should include a ‘review-by’ date.^20^ In practice, many WHO departments have taken 5 years as the norm for updating. For some individual recommendations, however, a rapidly changing evidence base may warrant more frequent updating. For example, the WHO recommendation on calcium supplementation during pregnancy has been updated four times in the past 10 years.^2 10 21 22^ When new evidence that could impact clinical decision-making becomes available, failing to promptly update the corresponding recommendation can adversely affect people’s health and well-being, and also undermine its credibility (ie, the degree to which the recommendation is considered by end users to be trustworthy and reflective of current evidence).

Conversely, many recommendations have seen little change in the underlying evidence base. For example, in 2011 WHO recommended the use of magnesium sulfate for preventing eclampsia in women with pre-eclampsia.^2^ The Cochrane review on this question (last published in 2010) demonstrated conclusively the benefits of this treatment, largely driven by the findings of the placebo-controlled, multicountry Magpie Trial involving over 10 000 women.^23 24^ Thus, the question on magnesium sulfate for eclampsia prophylaxis is considered ‘closed’ (from a benefits and harms standpoint)—updates of the systematic review of effectiveness will not alter the current recommendation because new trials on the same question would be unethical. In this situation, the recommendation remains up to date in terms of its evidence base, although it may be considered ‘old’ by end users due to the age of the evidence synthesis and publication.

Updating WHO recommendations is time-intensive and resource-intensive, requiring systematic collection and appraisal of the evidence related to benefits and harms, how stakeholders value the relevant outcomes, feasibility, acceptability, cost-effectiveness and implementation considerations. This evidence is then considered by an international panel of experts and stakeholders to formulate the recommendations.^20^ Identifying the recommendations that should be prioritised for updating would allow a more rational use of limited resources, while improving responsiveness to new, potentially important evidence. A new, dynamic system of prioritising and updating WHO MPH recommendations, combined with literature surveillance, has been developed and implemented, to respond rapidly to important changes in available evidence and ensure that accurate, relevant and up-to-date guidance is available to women, clinicians, policymakers and other stakeholders globally. In this article we describe the development and application of this approach to WHO global recommendations on MPH.

## Step 1: Establishing a WHO steering group and defining the scope of work

A WHO steering group (composed of staff from the Departments of Reproductive Health and Research and Maternal, Newborn, Child and Adolescent Health) was established to lead the development of this new ‘living guidelines’ approach to prioritising, updating and developing individual WHO MPH recommendations.^25^ As existing WHO recommendations in this area are largely (but not exclusively) derived from Cochrane systematic reviews, editorial staff from Cochrane Pregnancy and Childbirth were co-opted to form the Technical Working Group for this approach, working closely with the WHO steering group to collate, and appraise the status of evidence. The processes and methods used by the WHO steering group are consistent with international standards and best practices for guideline development, as recommended and implemented by the WHO GRC.^20^

The scope of work included several priority MPH topics, including health conditions (hypertensive disorders of pregnancy, postpartum haemorrhage (PPH), peripartum infections, preterm birth), interventions (induction of labour, augmentation of labour, caesarean section) and packages of care (antenatal care, intrapartum care, postnatal care and health promotion interventions). As of December 2016, WHO had published 10 guidelines (with 177 individual recommendations) on these topics.^1–10^ For each included topic, the working group considered both existing recommendation questions (ie, questions where a WHO recommendation already exists) and new questions (ie, questions that have been identified as critical to improving clinical practice or health programmes, but for which there have been no WHO recommendations previously). By question, we mean research questions structured using the Participant, Intervention, Comparison, Outcome format, however in a few instances we identified different types of research questions (such as the definition of a health condition).

## Step 2: Defining a framework and establishing an independent advisory group

**Figure 1:**
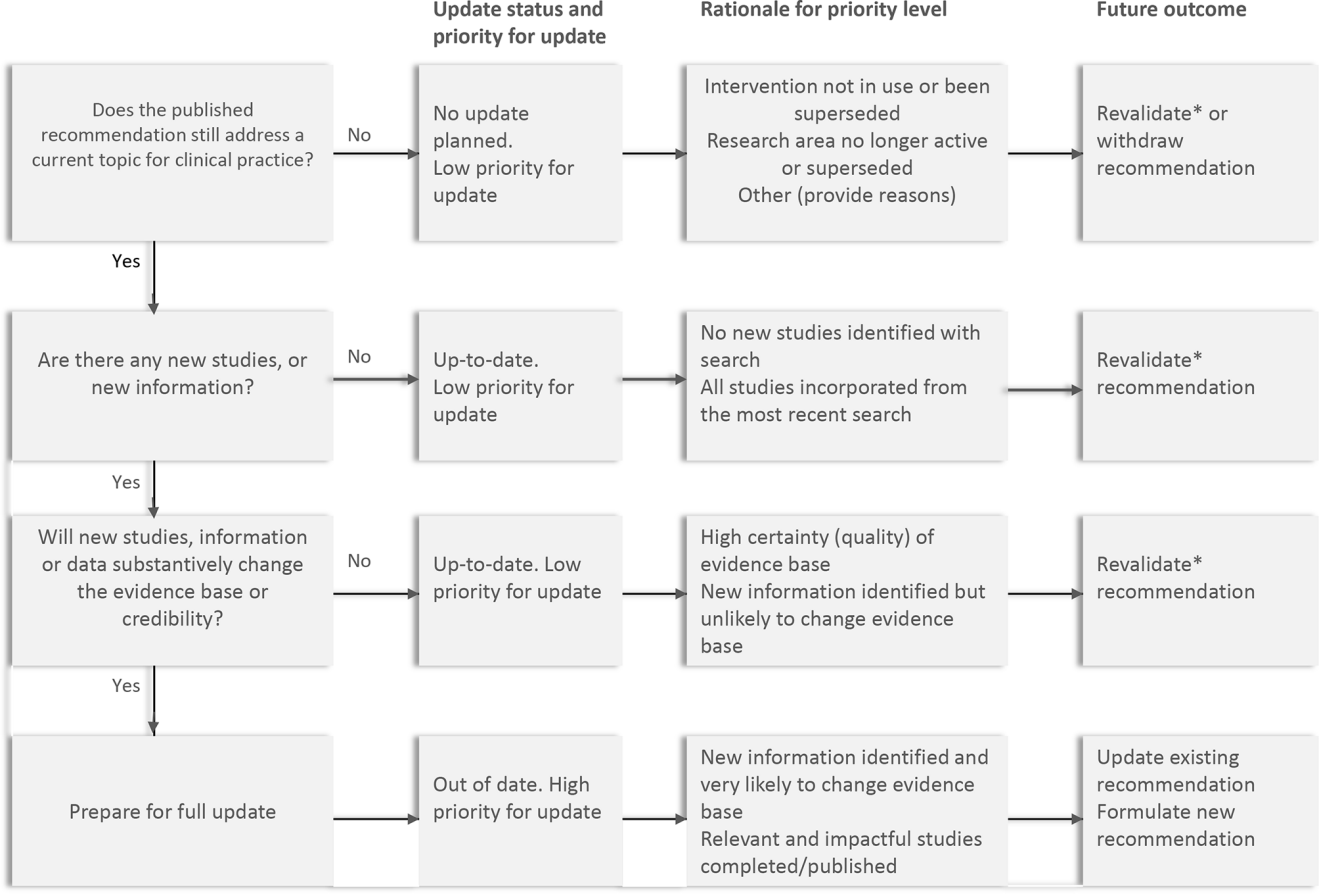
Framework for assessing priority for updating (adapted from Garner *et al* [29]). *By ‘revalidate recommendation’, we mean reaffirming the existing recommendation in terms of direction and technical content. The wording of the recommendation may be revised to improve clarity.

From March to October 2016, we conducted a focused literature search to identify existing frameworks and approaches relating to ‘living’ systematic reviews and guidelines.^26–30^ The WHO steering group drafted a framework (figure 1) as part of the standard operating procedures (SOPs) for operationalising the ‘living guidelines’ approach. These SOPs included description of the roles and responsibilities for the groups involved (online supplementary table 1) and how questions would be prioritised and updated. It was designed to be congruent with the WHO guideline development internal procedures and will be updated on an ongoing basis (available on request). The first draft of the document was prepared in October 2016 by the WHO steering group. Initial feedback was sought from the WHO GRC secretariat, WHO guideline developers in other thematic areas, guideline methodologists and members of the Cochrane Pregnancy and Childbirth editorial team. The SOPs were revised based on their input.

10.1136/bmjgh-2019-001683.supp1 Supplementary data

In December 2016, we established a new, independent Executive Guideline Steering Group on Maternal and Perinatal Health Recommendations (the Executive GSG) to play an advisory role in helping WHO maintain an up-to-date and balanced portfolio of recommendations.^31^ The 15 GSG members were selected to ensure a balance of geographical location, gender and expertise. The GSG includes maternal, newborn and health systems experts from the six WHO regions with no significant conflicts of interests. GSG members are content experts, skilled in the conduct and critical appraisal of evidence and in implementation of evidence-based recommendations at different health system levels. The SOPs were circulated to the GSG for feedback and comments were incorporated. We continued to develop the SOPs through its application to this first cycle of prioritising and updating (described below). The approach was also discussed and formally endorsed by the WHO GRC.

Figure 1 shows the framework underpinning the prioritisation and updating process as described in the SOPs, adapted from the framework for prioritising systematic reviews for update developed by Garner *et al*.^29^ It is composed of three critical decision points that the Executive GSG would use to assess whether an update is warranted (in step 4):

1. Does the published recommendation still address a current topic for clinical practice or programmes?
2. Are there any new studies or new information relating to the recommendation question?
3. Will any new studies, information or data substantively change the evidence base for the recommendation or the recommendation’s credibility?

## Step 3: Prioritising questions and mapping evidence

In February 2017, the WHO steering group conducted an international stakeholder prioritisation exercise via an online survey. The group developed a long list of stakeholders in maternal and newborn health across a range of disciplines, organisations and settings for potential participation, including clinicians, researchers, implementing organisations, professional associations, United Nations agency staff and consumer advocacy organisations. The survey was also circulated to the executive boards and national society presidents of two professional associations (the International Federation of Obstetricians and Gynaecologists and the International Confederation of Midwives). Some, but not all, of the individuals invited to participate had experience on WHO MPH guideline panels. The survey included the list of existing WHO MPH recommendations, and participants were asked to prioritise all questions as ‘low’, ‘medium’, ‘high’ priority for updating the recommendation. They were also asked to nominate any new questions they considered a priority. An ‘unknown’ option was available for situations where the survey participant felt they did not have sufficient expertise or information to make a ranking. Participants were asked to prioritise based on the following criteria which were developed to align with the framework in figure 1:

- The question is of public health importance (burden of suffering is high and expected effectiveness of the intervention in the question has the potential to reduce that burden).
- There is new evidence that has the potential to change the existing WHO recommendation.
- There is new evidence that could impact on the credibility of the existing recommendation.
- There is potential for an updated WHO recommendation to significantly change clinical practice globally.

­

Using the responses, a prioritisation score was calculated for each question (ie, percentage of survey respondents who ranked the question as a medium-priority or high-priority). For example, one question was ranked by 91 participants: 36, 37 and 16 participants ranked this question as low, medium and high priority, respectively (2 participants responded ‘unknown’). The prioritisation score ((37+16) / 91) was 58.2% for this question.

The online survey was conducted over a 6-week period in English only. A total of 264 stakeholders was invited to participate via email, with reminders at 2 weeks and 4 weeks. In total, 124 stakeholders participated (62% response rate), with all WHO regions represented (ranging from 5.6% of participants from South-East Asia to 28.2% participants each from Europe and the Americas) (table 1). Sixty-two participants (50.0%) described themselves as primarily an obstetrician/gynaecologist, 18 participants (14.5%) as a researcher, 15 participants (12.1%) as a nurse or midwife, and 10 (8.1%) as an epidemiologist. The remainder included neonatologists (8, 6.5%), policymakers (3, 2.4%), clients/patients (2, 1.6%), a reproductive health programme manager (1, 0.8%), funding agency staff (3, 2.4%) and staff of a professional association (2, 1.6%). Across all 177 questions included in the survey, the median of all prioritisation scores was 36.2% (range 14.1% to 73.6%). A total of 27 questions had a prioritisation score greater than 50%, and 6 questions had a prioritisation score greater than 60%.

**Table 1:** Characteristics of participants in the prioritisation survey

	Survey participants (N=124)	
	N	%
Age		
< 30 years	1	0.8
30–39 years	12	9.7
40–49 years	28	22.6
50–59 years	43	34.7
60 years or over	40	32.3
Which best describes you (pick one only)?		
Client or patient	2	1.6
Epidemiologist or public health specialist	10	8.1
Neonatologist	8	6.5
Nurse or midwife	15	12.1
Obstetrician/gynaecologist	62	50
Policymaker	3	2.4
Reproductive health programme manager	1	0.8
Researcher	18	14.5
Staff of a funding agency	3	2.4
Staff of a professional association	2	1.6
WHO region:		
Africa	25	20.2
Americas	35	28.2
South-East Asia	7	5.6
Europe	35	28.2
Eastern Mediterranean	8	6.5
Western Pacific	14	11.3

In parallel with the survey, the Technical Working Group (ie, the group performing mapping and surveillance of evidence for prioritisation) and the WHO steering group conducted an evidence mapping exercise for all recommendations underpinned by Cochrane reviews. The groups mapped the existing WHO MPH recommendations to their corresponding question and systematic review(s) of benefits and harms, and conducted new searches of the Cochrane Pregnancy and Childbirth’s Specialised Register of Controlled Trials to identify the number of new trial reports that had not yet been incorporated into the existing systematic review. For questions where a large number of new trial reports were identified, a brief, narrative overview of the findings of the trials with the largest sample sizes was prepared. These findings were summarised in a spreadsheet (along with the corresponding prioritisation scores) and shared with the GSG members ahead of the face-to-face meeting described below.

## Step 4: Selecting the high-priority questions for updating or developing WHO recommendations

In March 2017, a face-to-face meeting of the Executive GSG was held. Five observers from partner organisations were also invited to attend (representatives of professional associations, maternal and newborn health programmes, and funding agencies). The objective was to reach consensus on a short list of questions that should be prioritised for recommendation development or updating over the next 2 years.

At the meeting, the new approach was discussed and the GSG agreed on the definitions of priority levels they would assign to each question (table 2). Over the course of 2 days, the Chair facilitated a question-by-question assessment, informed by the prioritisation scores and evidence mapping. Questions often differed in the volume of evidence available, as well as the population, setting and health worker cadres they applied to. A set of ‘decision rules’ for identifying high-priority questions emerged from these discussions (box 1) and were used to operationalise the prioritisation framework presented in figure 1. The group reached consensus on a list of 36 high-priority questions (of which 6 were new questions) across different topics—intrapartum care (6 questions), peripartum infections,^2^ hypertensive disorders of pregnancy,^8^ induction of labour,^4^ PPH,^8^ antenatal care^3^ and health promotion.^5^ A spreadsheet detailing the 36 high-priority questions was published online on a WHO web page.^31^ A separate web page was also published, where stakeholders can nominate new priority MPH questions for the GSG to consider for future recommendations.^32^

Box 1 **Decision rules for prioritisation by the Executive Guideline Steering Group (GSG)**

- There is a substantial number of new effectiveness or other^1^ studies or reports in relation to the question, and these studies have not yet been assessed or included in a systematic review, particularly if the new data may relate to priority outcomes and or harms/safety. These new data may lead to changes in:
  - Estimates and CIs for specific outcomes.
  - Certainty of the evidence for specific outcomes.
  - Recommended dose or intensity of an intervention.
  - Recommended timing of an intervention (eg, gestational age).
  - Recommended subgroups for whom an intervention is effective (age groups, disease severity, etc).
  - Recommended route through which the intervention is delivered/administered.
  - Supporting interventions required.
  - Level of care/type of providers for delivering/administering the intervention.^2^
- There is a substantial number of new efficacy studies/reports in relation to the question, and these studies have not yet been assessed or included in a systematic review. Not including these studies may undermine the credibility of the recommendation, even if this is already a strong recommendation.
- There is a new drug or intervention in relation to a health issue, and data are now available.
- The recommendation is ‘general’ in relation to a class of drugs (eg, antihypertensives) and does not specify the use of a particular drug. There is now new evidence that suggests that the recommendation needs to be narrowed to a particular drug/s or suggests that particular drug/s are not effective or are less effective.
- The identification of definitional, consistency or interpretation issues in existing recommendations:
  - The definition of a clinical condition (eg, definition of ‘severe hypertension’) or of other relevant issues, and/or how the clinical condition is diagnosed^3^ or diagnostic criteria, have changed substantially since the recommendation was made.^4^
  - The wording of the recommendation is ambiguous, unclear or could be improved substantially (eg, in relation to drug dosages).
  - There are inconsistencies between recommendations (eg, in drug dosages, route of administration) that may lead to misunderstandings.
  - Guideline users in service delivery settings have indicated that the recommendation is hard to understand or interpret.
- There have been wide-scale changes in technologies (or access to technologies) that impact on clinical practice (eg, the viability of the fetus at particular gestational ages) and/or what life-saving interventions that can be delivered.
- A related recommendation has changed or has been prioritised for updating.
- New synthesis methods (such as network meta-analysis or individual patient data meta-analysis) may give new findings and/or allow comparisons and subgroup analyses that were not possible previously.
- The Executive GSG is of the view that a recommendation should be viewed as a good practice statement (or vice versa).
- The question underlying the recommendation needs to be revisited:
  - It is not clear if the question is still relevant.
  - The question is still relevant but needs to be unpacked or broken down further into subquestions. For example, a recommendation that health promotion is recommended needs to be broken down to consider which kinds of health promotion interventions are effective and can be recommended.
- Where the development of an existing recommendation was driven strongly by evidence related to how people value outcomes, or the acceptability, feasibility, equity or cost-effectiveness of the intervention, and there are changes (or potential changes) in the supporting evidence regarding these criteria.

1 For example, related to the acceptability, feasibility, equity or cost-effectiveness of the intervention.2 The GSG may flag recommendations that also have implications for changes to health systems recommendations.3 For example, if a clinical recommendation on a hypertensive drug is changed, WHO may also need to consider whether its recommendations on which cadre can deliver that drug also needs to be revised3 For example, new gold standard diagnostic test.4 Note that changes to definitions may have implications for multiple recommendations.

**Table 2:** Priority levels assigned to each question by the Executive Guideline Steering Group

High priority*	Based on new evidence that could potentially impact the current evidence base, the recommendation is out of date and a high priority for updating, or high priority for development of a new recommendation.
Low priority	New reports or information are unlikely to impact on current evidence base and no update is planned for this recommendation, or; An updated search indicates that zero new reports or information are available, the recommendation is up to date and can be revalidated.
Further assessment required	For those questions where the group felt they could not make an assessment of high or low priority without further information (such as the contents of new trial reports or locating possible additional reports).

*In some instances, updating high priority recommendations needs to be aligned with completion of an ongoing study that is expected to have a significant impact on current recommendation/s and/or clinical practice.

## Step 5: Developing recommendations with a ‘living’ guideline development group

After the meeting, the Cochrane Pregnancy and Childbirth editorial team initiated the creation or updating of Cochrane reviews on the effectiveness of interventions related to the high-priority questions and developed the corresponding evidence profiles. In order to help guideline panels make evidence-informed decisions, WHO staff developed GRADE evidence-to-decision (EtD) frameworks for each high-priority question.^33^ These frameworks have been used previously in WHO MPH guidelines, and allow explicit and transparent assessment of prespecified criteria (including desirable and undesirable effects, values and preferences, resource requirements, equity, acceptability and feasibility).^34^ Assessments of how stakeholders value the outcomes, and on equity, acceptability and feasibility, were informed by recent qualitative evidence syntheses on antenatal and intrapartum care.^35–37^ Where needed, new qualitative evidence syntheses were conducted.

In June 2017, the WHO steering group identified approximately 50 experts and stakeholders from the six WHO regions to constitute a WHO MPH ‘living’ guideline development group (GDG). This is a diverse pool of stakeholders skilled in critical appraisal, guideline development, implementation, clinical practice, policy and programmes relating to maternal and newborn health. The mix of experts and stakeholders was based on considerations of previous GDG membership for existing WHO MPH guidelines, while allowing for introduction of new members with specific expertise relating to content, programmatic area or guideline development methods. ‘Living guidelines’ panels were drawn from this pool by matching recommendations under consideration with individual expertise. Thus a group of approximately 15–20 individuals from the MPH GDG (with appropriate geographical and gender balance, and thematic expertise) was convened virtually on an as-needed basis.^25^ Partner organisations are invited to observe and contribute to guideline panel meetings, but are not permitted to vote (in the event a formal vote is required).^20^

In accordance with WHO guideline development standards, the GDG reviews the EtD frameworks, including the evidence profiles for benefits and harms, how stakeholders value outcomes, resource requirements, cost-effectiveness, acceptability and feasibility and the intervention’s impacts on equity. During the ‘living guidelines’ panel meeting, the GDG formulates the updated or new recommendations, and prepares clarifying remarks, implementation considerations and research priorities. These recommendations are peer reviewed by external experts, and then reviewed by the WHO GRC as part of the organisation’s quality assurance process prior to publication.

Between April 2017 and December 2018, 25 new or updated recommendations had been published based on the questions prioritised by the process described above.^12 21 38–42^ We briefly describe the updating of prioritised recommendations on PPH prevention and management as illustrative examples in boxes 2 and 3.^38^ All steps were conducted with a view to a sustained ‘living guidelines’ process of literature surveillance, prioritisation and updating of WHO maternal and perinatal recommendations beyond 2019.

Box 2 **Operationalising the ‘living guidelines’ approach: WHO’s recommendation on tranexamic acid (TXA) for the treatment of postpartum haemorrhage (PPH)**

TXA is a competitive inhibitor of plasminogen activation, and it can reduce bleeding by inhibiting the enzymatic breakdown of fibrinogen and fibrin clots.^59^ While it is widely used in trauma and surgery, at the time of the 2012 Guideline Development Group (GDG) meeting there was no direct evidence on the effectiveness and safety of TXA when used for the treatment of PPH. Consequently, in 2012 WHO conditionally recommended the use of TXA for the treatment of PPH only when uterotonics fail to control the bleeding, or when the bleeding is thought to be partly due to trauma.

On 26 April 2017, a large, randomised controlled trial—the World Maternal Antifibrinolytic (WOMAN) Trial—examining the effect of early treatment with TXA in women with PPH was published.^60^ Briefly, it was a randomised, double-blind, placebo-controlled trial, that randomised over 20 000 women in 21 countries with a clinical diagnosis of PPH to a regimen of intravenous TXA or identical placebo. The trial authors concluded that intravenous TXA reduces death due to bleeding in women with clinically diagnosed PPH, and that early treatment appears to optimise benefit. Aware of the forthcoming WOMAN Trial findings, the Guideline Steering Group prioritised this question for urgent updating. A new Cochrane review of antifibrinolytics for PPH treatment was rapidly initiated 61 as an offshoot of the existing Cochrane review on PPH treatments.^62^ The new review identified only two trials that compared the use of any fibrinolytic drug with no treatment in women with PPH, and findings were dominated by the WOMAN Trial.^60^ In addition, a new individual participant data meta-analysis of 40 138 patients was also available that demonstrated that early treatment of bleeding with TXA was effective, but delays in administration reduced effectiveness.^63^

On 29 August 2017, WHO convened an online GDG of 14 experts to review the evidence and revise the recommendation. On 31 October 2017, WHO published the updated recommendation on early use of intravenous TXA (within 3 hours of birth) in addition to standard care for women with clinically diagnosed PPH following vaginal birth or caesarean section.

Time from search for Cochrane systematic review to release of updated recommendation: 5 months, 3 days.

Box 3 **Operationalising the ‘living guidelines’ approach: WHO’s recommendations on uterotonics for the prevention of postpartum haemorrhage (PPH)**

Uterotonics (such as oxytocin) are routinely administered to all women during the third stage of labour to prevent PPH and its resulting complications. In 2012, WHO recommended oxytocin (10 IU, intravenous or intramuscular) as the uterotonic drug of choice, which was based on consideration of four Cochrane reviews of different uterotonic options; however, if oxytocin and/or skilled birth attendants are unavailable, other uterotonic options can be used.^3 64–67^ At its meeting in 2017, the Executive Guideline Steering Group prioritised updating these recommendations in anticipation of the results of the WHO-led PPH prevention trial that randomised nearly 30,000 women to a heat-stable formulation of carbetocin or oxytocin (the WHO CHAMPION Trial) and ongoing Cochrane systematic review with a network meta-analysis (NMA).^68^ This NMA included 140 trials assessing benefits and harms of all uterotonic options compared with placebo or each other for PPH prevention and was published on 25 April 2018.^69^

As CHAMPION and other trial results became available, WHO collaborated with the NMA authorship group for a rapid update that included all WHO prioritised outcomes and comparisons (leading to inclusion of an additional 56 trials). The updated review includes 196 trials involving 135 559 women.^70^ In parallel, WHO commissioned new systematic reviews on the views and experiences of women and healthcare providers, PPH prevention interventions and relevant uterotonic cost-effectiveness studies to inform relevant domains of evidence-to-decision frameworks for priority questions.^71 72^ WHO convened the PPH guideline panel for two virtual meetings (11-12 September and 3-4 October 2018) to review and update the relevant recommendations, which were published on 18 December 2018.^40 73^

Time from updated search of NMA to release of updated recommendations: 6 months, 24 days.

## Operationalising the living guideline approach

Guideline developers worldwide are facing the challenge of ensuring that recommendations are up to date in the context of a rapidly evolving or growing evidence base.^16 17^ Through this new ‘living guidelines’ approach, WHO can respond rapidly to new and important evidence without compromising the quality and rigour of its guideline development process. Implementing this approach adapts techniques already used in development of rapid advice guidelines (such as for public health emergencies), including early engagement with a standing guideline panel members and peer reviewers, and the use of virtual guideline panel meetings. The most compelling advantages of this approach are more rapid availability of updated recommendations and reduced costs through a process that maintains rigour and transparency. The average WHO MPH guideline (containing multiple recommendations) typically takes 1–2 years and considerable resources to develop. The efficiencies of the living guidelines approach are largely driven by, first, focusing efforts only on those individual recommendations where an update is warranted. This avoids the substantial costs associated with updating reviews and convening guideline panels, even where the strength or direction of a recommendation is unlikely to change. Second, the use of more frequent online GDG meetings, rather than in-person GDG meetings requiring international travels, also reduces costs and is more time-efficient for GDG members.

This new approach to dynamically updating individual WHO recommendations brings several new challenges. There is not yet clear consensus on the most efficient and effective methods for prioritising the recommendation questions that should be updated, and such standards are needed.^27 43^ While the prioritisation survey of international stakeholders helped inform the GSG’s discussions, the judgements of survey participants on a recommendation’s credibility or need for updating may be subjective. A systematic review by Vernooij *et al* reported that many organisations that issue guidelines do not provide adequate guidance on methods and procedures for updating.^44^ While we aimed to engage with a range of stakeholders in prioritising questions for updating, better engagement with those who will be affected by the recommendations (ie, women and their families) and elicitation of their perspectives and preferences are needed. The WHO steering group is exploring ways to better engage women in future prioritisation cycles.

Qualitative evidence syntheses on the views and perspectives of all stakeholders and consumers are being used increasingly in the development of WHO guidelines.^45^ These syntheses have proved critical to ensuring that the perspectives of women and their families are explicitly considered in WHO MPH recommendations.^36^ However, the scope of evidence surveillance and prioritisation of systematic reviews for rapid updating in this project has been largely informed by findings from quantitative systematic reviews on the effects of interventions. Further innovation is needed in order to apply a ‘living’ systematic review approach to qualitative evidence syntheses^46^ that may inform the EtD framework criteria on whether an intervention is acceptable to key stakeholders and whether an intervention is feasible to implement. Further work is also needed to apply a ‘living’ systematic review approach to other types of evidence that contribute to guideline development and decision-making in public health, such as cost-effectiveness studies, economic analyses or surveys on the views of key stakeholders.

Evidence mapping is itself a time-consuming process, particularly with a large number of recommendations. For a ‘living guidelines’ approach to work, guideline developers need to implement systems for continuous, systematic literature surveillance, rapid appraisal of the potential impact of new evidence and (where appropriate) rapid updating of systematic reviews.^25 47^ Use of machine learning for literature surveillance and evidence synthesis is being investigated which could make these processes more efficient in the near future.^48 49^ We plan to explore methods on how to rapidly assess whether systematic review update is warranted. Some authors have explored the use of statistical tests to assess the likelihood of whether new evidence will change the conclusions of a systematic review; however further research is required.^50–52^ The collaboration between WHO and Cochrane Pregnancy and Childbirth has proven essential to mapping of new trial reports in relation to Cochrane reviews, coordinating updates and preparing evidence profiles, as well as ensuring an agile response to new priorities. It should be acknowledged, however, that such collaboration does not exist across all WHO guideline priorities and technical departments and other mechanisms of maintaining a robust repository of evidence supporting WHO recommendations will be required to support ‘living guidelines’.

As Akl *et al* have emphasised, ‘living’ recommendations require ‘living’ systematic reviews, evidence profiles, EtD frameworks, guideline panels and budgets.^25^ To this we would add the need for a ‘living’ prioritisation process, in addition to ensuring that institutional policies and approval mechanisms can respond flexibly to updated recommendations. A 2017 systematic review by Martinez Garcia *et al* identified considerable variability in methods for prioritising updates of systematic reviews and guidelines; a prioritisation tool is under development.^53 54^ In the context of a large guideline portfolio and evidence base as in this case, a ‘living’ prioritisation process is critical to determining which systematic reviews demand resources for more frequent surveillance and possible updating using new technologies comprising both human and machine effort. It is important, however, to carefully examine the need for and implication of investing in living systematic reviews, even for a subset of prioritised recommendations. Switching systematic reviews to a ‘living’ mode is only worthwhile for questions where research remains active or there are lingering controversies, but less so in situations where questions have been prioritised for update based on considerations other than research evidence. While we maintained surveillance of the Cochrane Pregnancy and Childbirth database for new, impactful evidence, none of the Cochrane reviews underpinning this group of recommendations have yet transitioned to continuous evidence surveillance or to an updating model that combines human and machine effort.^47^ We plan to explore such a model to shorten the time required to prepare the evidence base for consideration by a guideline panel.

Updating individual recommendations also presents a challenge to dissemination, communication and subsequent implementation. WHO has a responsibility to ensure that recommendations on interventions that can impact health are made available to WHOMember States as soon as possible. However, it is likely that national healthcare decision-makers can only maintain a limited number of adoption and adaptation processes simultaneously, and these have not been developed with ‘living’ recommendations in mind. Prioritisation and updating processes would benefit from explicit consideration of the likely impact of updating recommendations on these stakeholders.

MPH recommendations have historically been packaged within a thematic guideline as a static paper or electronic document. Digital tools or platforms that can reflect the latest, up-to-date recommendations while linking to the broader guideline will be needed to disseminate those recommendations developed or updated within this new approach. For example, the WHO Reproductive Health Library now hosts the latest version of all WHO’s MPH recommendations, with a brief history of the recommendation’s previous iterations and planned updates.^55^ Other innovations (such as the MAGIC project’s interactive guideline platform, the *BMJ*’s Rapid Recommendations project and GRADEpro GDT) are also aimed at providing flexible, interactive digital platforms that can be easily updated if required.^56–58^

Policymakers and clinicians will need to be kept informed of important changes in individual recommendations on a more continuous basis, and implementation tools (such as job aids, checklists, decision support algorithms and the like) will also need to become more responsive to recommendation updates. Digital platforms that can connect guideline development and publication through to dissemination and implementation should be developed, to facilitate knowledge transfer to and uptake by end users.

## Conclusions

Developing and updating WHO MPH recommendations are now driven by a systematic and continuous process of prioritisation and evidence synthesis where the unit of update is individual recommendation rather than thematic guideline. This entails a shift towards a more efficient, responsive process that reflects the latest evidence, and leads to recommendations that optimise programmes and practice.
