# Supplementary material for: Developing and applying a 'living guidelines' approach to WHO recommendations on maternal and perinatal health
Source: BMJ Glob Health. 2019 Aug 19;4(4):e001683. doi: 10.1136/bmjgh-2019-001683 (PMC6703290; doi:10.1136/bmjgh-2019-001683)
Supplement: Supplementary data [file bmjgh-2019-001683supp001.pdf]

SUPPLEMENTARY TABLE 1: Main groups involved in the living guideline process

WHO Steering Group	Comprises WHO staff members from the Departments of Reproductive Health and Research (RHR); and Maternal, Newborn, Child and Adolescent Health (MCA), that guide and oversee the guideline updating process.
Technical Working Group	Composed of editorial staff from Cochrane Pregnancy and Childbirth and other relevant Cochrane Groups that work closely with the WHO steering group to collate and appraise status of evidence.
Executive Guideline Steering Group	Independent panel of 15 maternal, newborn and health systems experts and relevant stakeholders from the six WHO regions, with no significant conflicts of interest. Executive GSG members play a leadership/governance role in overseeing guideline updating procedures, supporting WHO in the following tasks:<br>• Prioritize key recommendation questions that need to be updated;<br>• Identify and recommend new questions that should be addressed within the context of updating a thematic area already covered by existing WHO guidelines;<br>• Define scope for new guideline thematic areas, including specific issues that need to be addressed in future guidelines<br>• Support WHO in maintaining an up-to-date and balanced portfolio of evidence-based recommendations in maternal and perinatal health.
WHO Maternal and Perinatal Health Guideline Development Group	A diverse pool of stakeholders who are skilled in critical appraisal, guideline development, implementation, clinical practice, policy and programmes relating to maternal and newborn health. The overall function of this group is to support WHO in continuously maintaining an up-to-date portfolio of evidence-based guidelines in maternal and perinatal health. Specifically, members of the group will perform the following tasks:<br>• Provide input into the scoping and drafting of the initial list of recommendations questions to be prioritized for update;

	• Participate in electronic surveys to prioritize questions that will guide evidence reviews for the update;<br>• Participate in virtual technical consultations to appraise and interpret evidence and consider other relevant issues to revise or revalidate recommendations; and<br>• Review final draft and approve updated or new recommendations for executive approval of WHO GRC and other WHO internal approvals<br><br>"Living guideline" panels are drawn from this pool by matching recommendations under consideration with individual expertise. Thus, a group of approximately 15 to 20 individuals from the MPH GDG (with appropriate geographic and gender balance, and thematic expertise) are convened virtually on an as-needed basis.
Partner Organizations (Meeting Observers)	Stakeholders for dissemination and implementation of updated recommendations are invited to attend and observe the Executive GSG and any MPH-GDG meetings. Representatives of professional societies (such as the International Federation of Gynaecology and Obstetrics (FIGO), International Confederation of Midwives (ICM), United States Agency for International Development (USAID), and Bill and Melinda Gates Foundation (BMGF)) as well as representatives of implementing organizations serve as observers. Observers are welcome to contribute to discussions of the Executive GSG, but (in the event a formal vote is needed) do not vote.
